# The core role of macrophages in hepatocellular carcinoma: the definition of molecular subtypes and the prognostic risk system

**DOI:** 10.3389/fphar.2023.1228052

**Published:** 2023-08-24

**Authors:** Qiaona Wang, Yunshou Lin, Wenguan Yu, Xiaogang Chen, Qingqing He, Zhiyu Ye

**Affiliations:** ^1^ Department of Breast Surgery, The First Affiliated Hospital of Ningbo University, Ningbo, China; ^2^ Department of Hernia and Hepatobiliary Surgery, The First Affiliated Hospital of Ningbo University, Ningbo, China

**Keywords:** hepatocellular carcinoma, macrophage, molecular subtype, random forest, prognosis, immunotherapy, chemotherapy, targeted drugs

## Abstract

**Background:** In patients with hepatocellular carcinoma (HCC), the tumor microenvironment (TME) is resistant to immunotherapy because of its specificity. It is meaningful to explore the role of macrophage, which is one of the most abundant immune cells in the TME, in cellular communication and its effect on the prognosis and immunotherapy of HCC.

**Methods:** Dimensionality reduction and clustering of the single-cell RNA-seq data from the GSE149614 dataset were carried out to identify the cellular composition of HCC. CellChat was used to analyze the communication between different cells. The specifically highly expressed genes of macrophages were extracted for univariate Cox regression analysis to obtain prognostic genes for HCC cluster analysis, and the risk system of macrophage-specifically highly expressed genes was developed by random forest analysis and multivariate Cox regression analysis. Prognosis, TME infiltration, potential responses to immunotherapy, and antineoplastic drugs were compared among molecular subtypes and between risk groups.

**Results:** We found that HCC included nine identifiable cell types, of which macrophages had the highest communication intensity with each of the other eight cell types. Of the 179 specifically highly expressed genes of macrophage, 56 were significantly correlated with the prognosis of HCC, which classified HCC into three subtypes, which were reproducible and produced different survival outcomes, TME infiltration, and immunotherapy responses among the subtypes. In the integration of four macrophage-specifically highly expressed genes for the development of a risk system, the risk score was significantly involved in higher immune cell infiltration, poor prognosis, immunotherapy response rate, and sensitivity of six drugs.

**Conclusion:** In this study, through single-cell RNA-seq data, we identified nine cell types, among which macrophage had the highest communication intensity with the rest of the cell types. Based on specifically highly expressed genes of macrophage, we successfully divided HCC patients into three clusters with distinct prognosis, TME, and therapeutic response. Additionally, a risk system was constructed, which provided a potential reference index for the prognostic target and preclinical individualized treatment of HCC.

## Introduction

The liver is a crucial organ with fundamental metabolic and immunological activities that sits at the crossroad of confluence of intestinal and systemic blood circulation, making it a prime location for multi-factor organ interactions (Kohlhepp et al., 2023). Approximately 844 million people worldwide are estimated to suffer from liver disease (Ramachandran et al., 2020). With a mortality-to-morbidity ratio of 0.95 (Chen et al., 2020), liver cancer is the most dangerous form of liver illness, and hepatocellular carcinomas (HCCs) are the most diagnosed malignancies of liver origin. The “trilogy pattern” describes how HCC develops and is characterized by cirrhosis, hepatitis B, and liver cancer (Liao et al., 2023). HCC is difficult to diagnose because symptoms do not occur until advanced stage or distant metastasis. Therefore, patients do not respond well to treatment, and cell diversity and complexity are believed to be key factors leading to treatment failure and fatal outcomes.

Using bulk RNA sequencing data, biomarkers for the diagnosis and prognosis of HCC are screened. For example, in a pan-cancer genomic study, PHF19 was uncovered to be a carcinogenic factor for HCC (Zhu et al., 2021). Several complement genes (C1R, C6, C7, CFP, and CFHR3) were also identified to be prognostic biomarkers in HCC patients (Qian et al., 2022). Moreover, immune- (Xin et al., 2022) and ferroptosis-related (Lin and Yang, 2022) long non-coding RNAs were also reported to be prognostic indicators for HCC. Notwithstanding great progress in distinguishing biomarkers based on bulk RNA sequencing data, these findings focused on mixed cells of HCC tissues and detected only an average gene expression level of mixed cells.

One of the most accurate ways to determine cell identification, status, function, and reaction is to examine the activity of its genes. At the transcriptome level, single-cell RNA sequencing (scRNA-seq) analysis offers a way to categorize, describe, and distinguish each cell (Jovic et al., 2022). In recent years, scRNA-seq technology has been increasingly applied in HCC studies, which has been used to analyze individual cells in tumor cells, tumor stem cells, and the tumor microenvironment (TME) (Zheng et al., 2018; Sun et al., 2021). As one of the main drivers of tumor heterogeneity, the TME is acknowledged as a highly dynamic network throughout cancer incidence, progression, and prognosis, as well as therapeutic treatments (Zhou et al., 2021). Tumor-associated macrophages (TAMs), as one of the most numerous immune cells invading the TME, are present at all stages of HCC development and play a crucial role as coordinators of disease course. TAMs play a critical role in the immune response and disease evolution, from benign tumors to malignant tumors, promoting angiogenesis immunosuppression, treatment resistance, and metastasis (Kohlhepp et al., 2023; Zheng et al., 2023). Presently, by eliminating existing TAMs, blocking TAM recruitment, reprogramming TAM polarization, regulating TAM products, and restoring TAM phagocytosis, targeted TAM therapy for HCC has achieved promising results (Xu et al., 2022). Qu et al. (2022) proposed a prognostic signature model applying M2-like macrophage-related biomarkers. However, comprehensive macrophage-related preclinical models are still needed to identify macrophage-targeted therapy.

In this study, we detected the cellular composition of HCC by scRNA-seq analysis, defined the type of HCC, constructed a risk system according to the specifically highly expressed genes of macrophages, and used it for prognosis assessment, immunotherapy response prediction, and drug screening, which provided clues for further clinical research of TAMs as a potential therapeutic direction of HCC.

## Materials and methods

### Download and preprocessing of scRNA-seq and RNA-seq data

The scRNA-seq dataset and RNA-seq dataset of HCC were downloaded by accessing the Gene Expression Omnibus (GEO) database (http://www.ncbi.nlm.nih.gov/geo/), and the scRNA-seq dataset was numbered GSE149614. There were two HCC RNA-seq datasets from the GEO database, GSE76427 and GSE14520. Other available RNA-seq datasets for HCC include TCGA-LIHC (https://portal.gdc.cancer.gov/) and HCCDB18. In addition, RNA-seq data and prognostic information about the immunotherapy cohort IMvigor210 (bladder cancer) (http://research-pub.gene.com/IMvigor210CoreBiologies), GSE91061 (melanoma), GSE135222 (non-small-cell lung cancer), and GSE78220 (melanoma) datasets were obtained. For the GSE149614 dataset, the following quality control indicators were used to eliminate gene expression interference in low-quality cells: 200 < total number of expressed genes per cell (nGenes); 200 < total number of UMIs per cell; percentage of unique molecular identifiers (UMIs) mapped to mitochondrial genes (MT%) < 10%; and unit read counts the ratio of the number of genes (log10GenesPerUMI) > 0.8. For RNA-seq datasets, samples that lack clinical follow-up information and survival data were removed, and for genes with multiple probes (for GEO data) or transcripts (for TCGA data), the median expression was used as the expression level for analysis.

### Dimension reduction and clustering of scRNA-seq data

Seurat includes a variety of built-in functions for dimensionality reduction and clustering of scRNA-seq data (Hao et al., 2021). First, to eliminate technical noise or bias and ensure comparability between each unit, the log-normalization function was used for standardization. The feature subset that shows high intercellular variation in the dataset is also called a highly variable gene (HVG), and its quality greatly impacts the accuracy of clustering. The FindVariableFeatures function in Seurat was used to detect the HVG. The samples were integrated, and the FindIntegrationAnchors function found the integration anchors. The IntegrateData function converted the anchor information into an integrated expression matrix. Before clustering, principal component analysis (PCA) is required for dimension reduction, which can not only reduce the indicators to be analyzed but also retain the original data information as much as possible (Pei et al., 2023). The clustering of cells was mainly based on two functions: FindNeighbors and FindClusters. Biological annotation in each cluster was examined to serve the basis for follow-up analysis (Jovic et al., 2022). The cluster was marked by automatic annotation through CellMarker 2.0 and manual annotation according to related studies (Peng et al., 2019; Su et al., 2021).

### Analysis of intercellular communication

Intercellular communication networks from scRNA-seq data can be quantitatively inferred, analyzed, and visualized using CellChat (Jin et al., 2021). The gene expression data of different cell types identified in GSE149614 were input into CellChat (Chi et al., 2023a), and the CellChatDB.human file was used as a reference to generate a network map of the number and intensity of interactions between cells.

### Clustering of HCC was performed by identifying macrophage-specifically highly expressed genes

The specifically highly expressed genes of macrophages identified in GSE149614 were extracted, and univariate Cox regression analysis was carried out according to their expression in the TCGA-LIHC cohort. Prognostic factors were selected for consensus clustering analysis in ConsensusClusterPlus (Wilkerson and Hayes, 2010). The cumulative distribution function (CDF) curve, delta area curve, and consensus matrix were generated for different k-values to demonstrate the optimal clustering effect (Yuan et al., 2022).

### Analysis of the genome variation map among subgroups

Mutated data in MAF format exported by TCGA’s mutect2 was processed using the “maftools” package in the R package (Miao et al., 2022). The total number of mutations in the sample was measured, and the genes with mutation number > 3 were identified. The high-frequency mutation genes of subgroups were screened by Fisher’s exact test and could be viewed as a waterfall map. Niknafs et al. discovered that immune checkpoint blockade treatment response is correlated with persistent tumor mutation burden (pTMB), which includes mutations in single-copy areas and those present in multiple copies per cell (Niknafs et al., 2023). pTMB was calculated and compared between subgroups according to different calculation methods.

### Immune and stromal fraction analysis of the tumor microenvironment

The cell types that make up HCC were identified in GSE149614, and the differences in the content of the identified cell types among macrophage subgroups were evaluated in the TCGA-LIHC cohort. The total infiltrating stromal cell scores and total immune cell scores in the TME were calculated using ESTIMATE (Yoshihara et al., 2013). Based on RNA-seq data, MCP-counter (Becht et al., 2016) inferred the absolute infiltration abundance of eight immune cells and two stromal cells. A previous study provided a way to calculate a comprehensive view of the immune landscape in the TME, immunophenoscore (Charoentong et al., 2017), in which the levels of infiltration of 28 types of immune cells in HCC samples were assessed.

### Screening prognostic model gene indexes from macrophage-specifically highly expressed genes using the machine learning method

Random forest is a compositional supervised learning method. “RandomForestSRC,” developed by Ishwaran and Kogalur (2016), calculated the importance of each gene during the training of macrophage-specifically highly expressed genes and ranked them from high to low and then chose the genes. Then, the stepAIC function of MASS helps eliminate the genes that cause multiple collinearities, and the remaining genes became the indexes of the prognostic model, and the equation was $Risk\;score=\sum_{i=1}^{n}\left({Coef}_{i}\times {\mathrm{Exp}}_{i}\right)$ .

Here, “Coef” and “Exp” refer to the Cox regression coefficient and expression level of the gene, respectively.

### Prognostic performance evaluation of the risk model

A prognostic model was used to quantify the risk score of samples in the training set (TCGA-LIHC cohort) and three independent verification sets (HCCDB18, GSE76427, and GSE14520). The “survminer” package divided the risk group for each cohort according to the risk score and generated a Kaplan–Meier curve (Chen et al., 2022). The “survivalROC” package generated a time-dependent receiver operating characteristic (ROC) curve based on the risk score (Dong et al., 2023). The closer the area under the ROC curve (Hao et al., 2021) is to 1, the more accurate the model is in predicting prognosis.

### Pathway correlation analysis of the risk score

Single-sample gene set enrichment analysis (ssGSEA) calculated the enrichment score for each sample paired with a gene set (Chi et al., 2023b). The h.all.v7.4.symbols.gmt gene set and 13 core biological pathway gene signatures were used here, the former obtained from the Molecular Signatures Database (MSigDB) (Liberzon et al., 2015) and the latter from the study by Mariathasan et al. (2018). The correlation between the gene set membership score or pathway enrichment score and the risk score was defined by Pearson’s correlation analysis.

### Analysis of immunotherapy response

Tumor Immune Dysfunction and Exclusion (TIDE) was produced to predict the potential response to immunotherapy; the TIDE score was calculated, which consists of two parts, dysfunction score and exclusion score, and the levels of the two parts are usually negatively correlated in cancer (Jiang et al., 2018). When the immune dysfunction gene has a higher weight, the weight of the immune dysfunction gene and the respective expression amount are multiplied and then added together to obtain the dysfunction score. The exclusion score was summed up by multiplying the expression of the exclusion genes with higher weight.

### Drug sensitivity prediction

The “pRRophetic” package (Geeleher et al., 2014) used the gene expression matrix adopted the linearRidge function of the Ridge package through the internal algorithm to carry out the ridge regression analysis to complete the prediction of drug sensitivity and further combined with the sample grouping file to find the drugs with different sensitivities under different groups.

### Cell culture and transfection

HCC cells (Hep3B and Huh7) were purchased from the Typical Culture Reserve Center of China (Shanghai, China), and human hepatocytes (THLE-2) were purchased from Cellcook Biotech Company (Guangzhou, China). Hep3B and Huh-7 cells were cultured in DMEM (Gibco, United States), while THLE-2 cells were cultured in BEGM (Gibco, United States) supplemented with fetal bovine serum (Gibco, United States) and penicillin/streptomycin at 37°C under 5% CO_2_. The negative control small interfering NC (si NC), PPT1 siRNA, and SAT1 siRNA (Sagon, China) were transfected into the cells utilizing Lipofectamine 2000 (Invitrogen, United States). The primer sequences for PPT1 siRNA and SAT1 siRNA are listed in Table 1.

**TABLE 1:** Primer sequences for PPT1 siRNA and SAT1 siRNA.

Gene	Primer sequence (5′-3′)
si PPT1#1	CTG​TTG​CAA​TCC​CTT​AAG​CAT​GG
si PPT1#2	CTG​GAA​TTT​ACG​TCT​TAT​CTT​TA
si SAT1#1	ATG​GAA​GAA​CAA​GTA​ATC​TTA​AC
si SAT1#2	TGG​AAG​AAC​AAG​TAA​TCT​TAA​CT

### Quantitative real-time polymerase chain reaction

TRIzol (Thermo Fisher, United States) reagent was used to extract the total RNA from Hep3B, Huh-7, and THLE-2 cell lines. cDNA was created from 500 ng of RNA using the HiScript II SuperMix (Vazyme, China). The PCR amplification conditions comprised 46 cycles of 94°C for 10 min, 94°C for 10 s, and 60°C for 45 s. GAPDH acted as the internal reference. The primer sequences for target genes are listed in Table 2.

**TABLE 2:** Primer sequences for PPT1, DAB2, FTL, SAT1, and GAPDH.

Gene	Forward primer sequence (5′-3′)	Reverse primer sequence (5′-3′)
PPT1	GGC​GTA​CTC​CAA​AGT​TGT​TCA​GG	CTG​CCA​AGA​AGA​TGC​TGT​GGT​TG
DAB2	CTC​TGT​CCA​GTC​CTC​ACC​ACA​T	GTT​CTG​AGA​CGG​GAG​GAG​CAA​A
FTL	TAC​GAG​CGT​CTC​CTG​AAG​ATG​C	GGT​TCA​GCT​TTT​TCT​CCA​GGG​C
SAT1	TAC​CAC​TGC​CTG​GTT​GCA​GAA​G	CTT​GCC​AAT​CCA​CGG​GTC​ATA​G
GAPDH	CTG​GGC​TAC​ACT​GAG​CAC​C	AAG​TGG​TCG​TTG​AGG​GCA​ATG

### Cell viability detection

Cell viability was detected using the Cell Counting Kit-8 assay (Beyotime, China). Cells from different treatments were cultured in 96-well plates at a density of 1 × 10^3^ cells per well. CCK-8 solution was applied at the indicated time points. After incubation at 37°C for 2 h, the OD 450 values of each well were detected using a microplate reader (Thermo Fisher, United States).

### Statistical analysis

All statistical analyses and visualizations were implemented by using R software. The statistical tests used included Student's t-tests, Fisher’s exact test, chi-square test, and Kruskal–Wallis test. For all statistical results, *p* < 0.05 was regarded as a significant difference, marked with *.

## Results

### Cellular composition and intercellular communication in HCC

**FIGURE 1:**
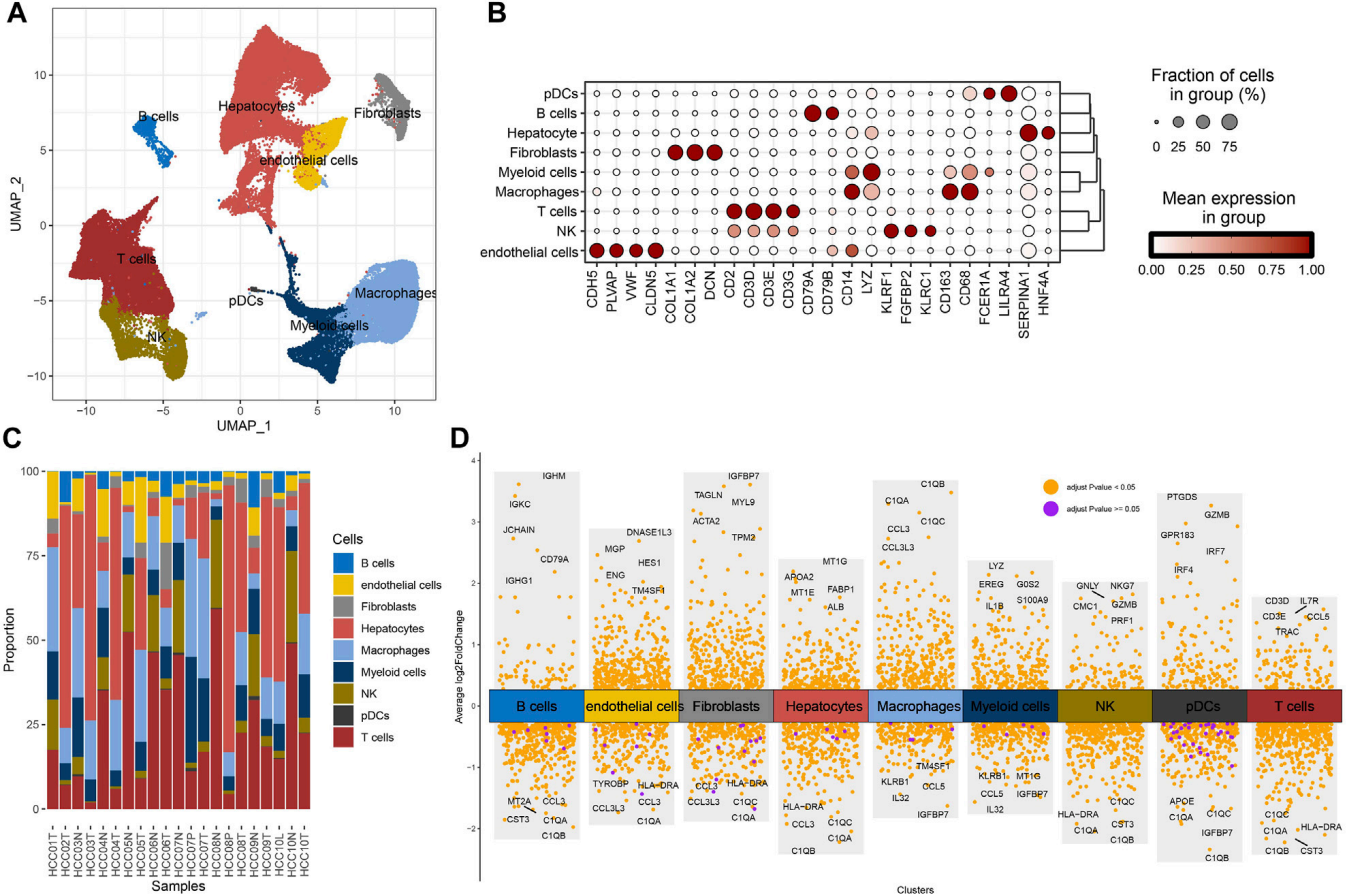
Cellular composition of HCC. **(A)** Uniform Manifold Approximation and Projection (UMAP) visualizes the distribution of cell types in GSE149614. **(B)** The distribution proportion and expression level of specific genes in each type of cell. **(C)** The proportion of cell types in each tissue sample in GSE149614. **(D)** The volcano map shows the differential marker genes for each type of cell.

To study the cellular composition of HCC, single-cell transcriptomes for 22 samples from four relevant sites from GSE149614 were collected, and 63,977 cells were reserved for differentiation after quality control. Unsupervised cell clustering revealed nine cell types based on the expression of lineage-specific marker genes: hepatocytes, B cells, fibroblasts, endothelial cells, T cells, plasmacytoid dendritic cells (pDCs), myeloid cells, NK cells, and macrophages (Figure 1A). Each cell type contained specific highly expressed genes, such as CDH5, PLV AP, VMF, and CLDN5 to endothelial cells; CD2, CD3D, CD3E, and CD3G to T cells; COL1A1, COL1A2, and DCN to fibroblasts; CD14, CD163, and CD68 to macrophages; CD79A and CD79B to B cells; LYZ to myeloid cells; KLRF1, FGFBP2, and KLRC1 to NK cells; FCER1A and LILRA4 to pDCs; and SERPINA1 and HNF4A to hepatocytes (Figure 1B). The composition proportion of cell types in tissue samples showed that the distribution proportion of nine kinds of cells in each sample was different; hepatocytes, T cells, and macrophages were the main cell types of HCC (Figure 1C). FindAllMarkers also helped identify specifically highly expressed genes for each type of cell (Figure 1D). According to the results of CellChat analysis and visualization, the communication intensity between the nine kinds of cells was determined. By disassembling the interaction between each cell and the other eight kinds of cells, it was found that the cell with the highest intensity of communication with B cells, endothelial cells, myeloid cells, NK cells, and pDCs was macrophages. At the same time, macrophage was also one of the cells with the strongest communication with fibroblasts, hepatocytes, and T cells (Supplementary Figure S1). These results reflected the important role of macrophage in HCC.

### Three subtypes of HCC were defined according to the specifically highly expressed gene of macrophage

**FIGURE 2:**
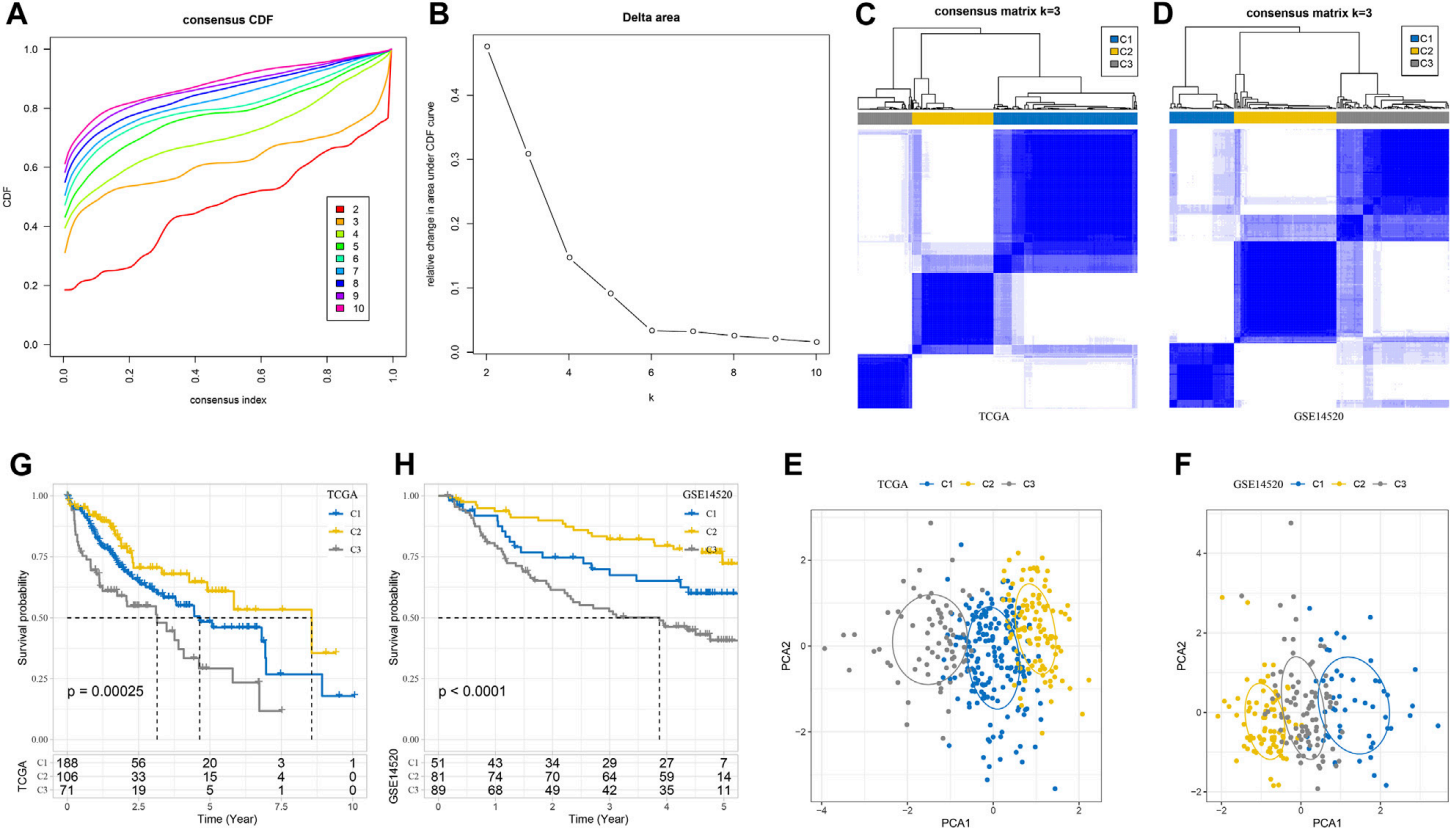
Three subtypes of HCC were defined according to the marker gene of macrophage. **(A)** CDF when k takes different values. **(B)** Consensus clustering delta area curve for each category number k compared with K–1. **(C)** A consensus clustering heatmap dividing the sample in the TCGA-LIHC cohort into three subgroups. **(D)** The heatmap for generating three clusters in the GSE1452 cohort when k takes 3. **(E)** PCA shows the different distributions of samples in TCGA-LIHC when three clusters are divided. **(F)** The PCA of GSE14520 cohort shows the distribution of the sample. **(G, H)** Subtype survival trends in the TCGA-LIHC cohort and GSE14520 cohort. The significance of the difference was marked with *, *p < 0.05, and ns, no difference.

A total of 179 specifically highly expressed genes were filtered in macrophage, and their prognostic significance in the TCGA-LIHC dataset was calculated by univariate Cox regression analysis. A total of 56 genes were identified as prognosis-related genes. The types of samples in the TCGA-LIHC dataset were defined according to the expression of 56 genes; k-values that could not simultaneously meet the CDF decline were not so drastic, and the CDF value which was not too small was found. The optimal k value that met the criteria was initially set as 3 (Figures 2A, B). A consensus clustering heatmap showed the clustering of the samples in the TCGA-LIHC cohort and GSE14520 cohort at *k* = 3, and it seems reasonable to divide the HCC samples in these two cohort groups into three categories (Figures 2C, D). The different distribution of samples when three clusters were divided was observed through PCA, which further confirmed the reliability of dividing the TCGA-LIHC cohort and GSE14520 cohort into three subtypes (Figures 2E, F). The subtypes of the TCGA-LIHC cohort and GSE14520 cohort had the same survival trend. At any time, C2 had the greatest chance of survival, C3 had the least chance of survival, and C1 had a greater chance of survival than C3 and less than C2 (Figures 2G, H).

### Genomic alterations and clinical features of three macrophage-related subtypes

**FIGURE 3:**
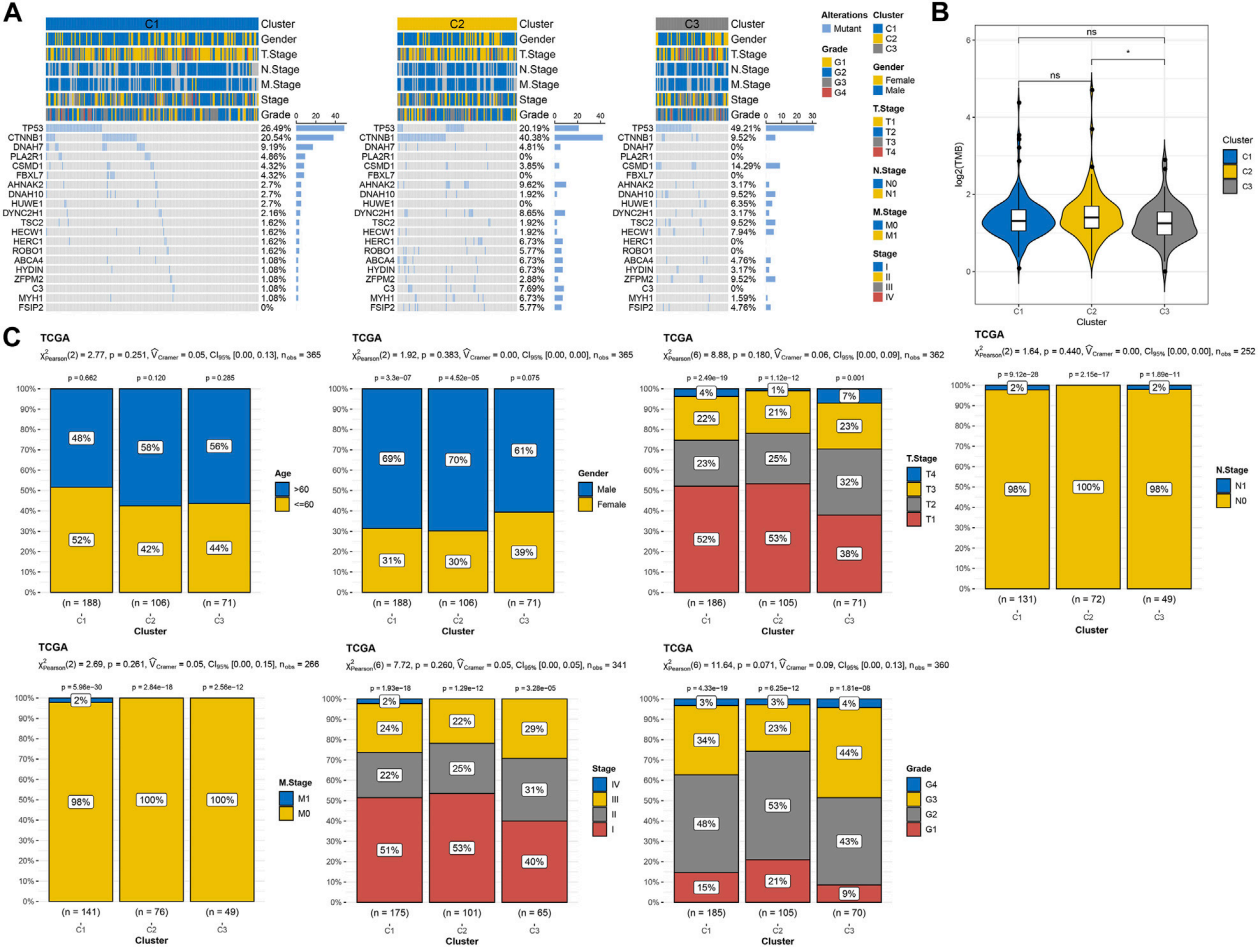
Genomic alterations and clinical features of three macrophage-related subtypes. **(A)** The waterfall map shows the mutation rates of the 20 genes with the highest mutation rates in three macrophage-related subtypes. **(B)** PTMB differences among three macrophage-related subtypes. **(C)** Age, gender, T stage, N stage, M stage, and stage and grade distribution among three macrophage-related subtypes. The significance of the difference was marked with * and ****p < 0.0001.

The differences of three macrophage-related subtypes were compared in terms of genomic characteristics and clinicopathological features. A total of 39 high-frequency mutant genes showed significant differences in mutation rates among three macrophage-related subtypes, among which the mutation rate of TP53, the most common mutation gene in human cancer, was 26.49% in C1, 20.19% in C2, and 49.21% in C3. The gene with the highest mutation rate in C2 was CTNNB1 (40.38%), and the mutation rate in C1 and C3 was 20.54% and 9.52%, respectively. The gene PLA2R1 with the third highest mutation rate in C1 had a mutation rate of 4.81% in C2, but no mutation was found in C3 (Figure 3A). Among the three macrophage-related subtypes, the pTMB of C2 with the best prognosis was significantly higher than that of C3 with the worst prognosis (Figure 3B). The characteristics of N stage, M stage, stage and grade, age, sex and, T-stage distribution were similar among the three macrophage-related subtypes without statistical difference (Figure 3C).

### Discriminations in signaling pathways and immunological features of three macrophage-related subtypes

**FIGURE 4:**
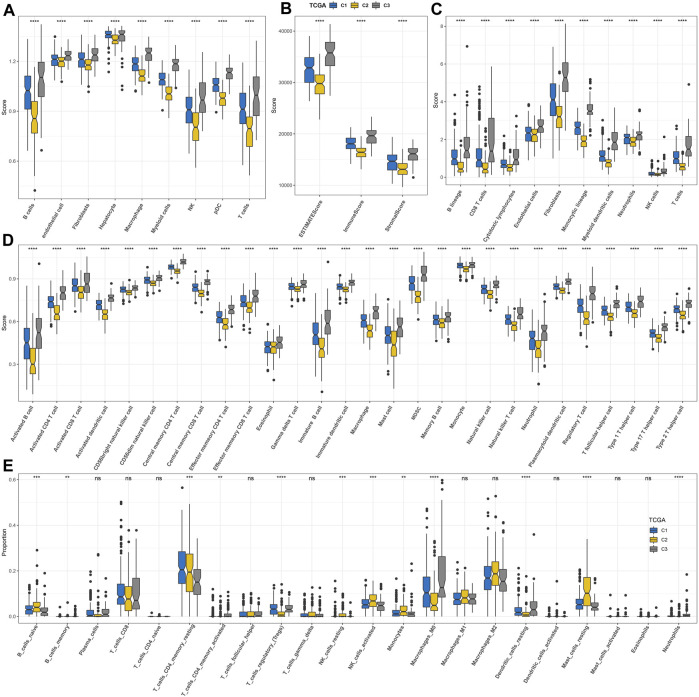
Discriminations in signaling pathways and immunological features of three macrophage-related subtypes. **(A)** Differences in the distribution of nine kinds of cells among three macrophage-related subtypes identified by scRNA-seq analysis. **(B)** ESTIMATE analysis. **(C)** MCP-counter analysis. **(D)** ssGSEA analysis. **(E)** CIBERSORT analysis. The significance of the difference was marked with *, *p < 0.05, **p < 0.01, ***p < 0.001, ****p < 0.0001, and ns, no difference.

By analyzing the enrichment of different biological pathways among subtypes, it was clearly found that C2 was the subtype most significantly negatively correlated with tumor-promoting signal pathways (such as TGF-β signaling, PI3K-AKT mTOR signaling, KRAS signaling up, and epithelial–mesenchymal transition) and immune activation pathways (such as inflammatory response, complement, interferon alpha response, and interferon gamma response) among three macrophage-related subtypes (Supplementary Figure S2). The nine kinds of cells identified in HCC also showed different distribution contents among three macrophage-related subtypes. The relative content of these nine kinds of cells in C2 was the least, while that in C3 was the highest (Figure 4A). The ESTIMATE score, immune score, and stromal score also showed significant differences among the three macrophage-related subtypes. The trend of the three indexes in the three macrophage-related subtypes was the same, which was the lowest in C2 and the highest in C3 (Figure 4B). Consistent with the results of ESTIMATE evaluation, the immune cells and stromal cells evaluated by MCP-counter and ssGSEA also showed different abundance levels in the three macrophage-related subtypes, with the lowest abundance in C2 and the highest abundance in C3 (Figures 4C, D). Additional CIBERSORT analysis illustrated the difference in macrophage subtypes with the highest score of macrophages_M0 in the C3 cluster and the lowest in the C2 cluster (Figure 4E). Immunological features were carried out in three clusters of the GSE14520 cohort with similar findings (Supplementary Figure S3).

### Different responses of three macrophage-related subtypes to immunotherapy and anti-tumor drugs

**FIGURE 5:**
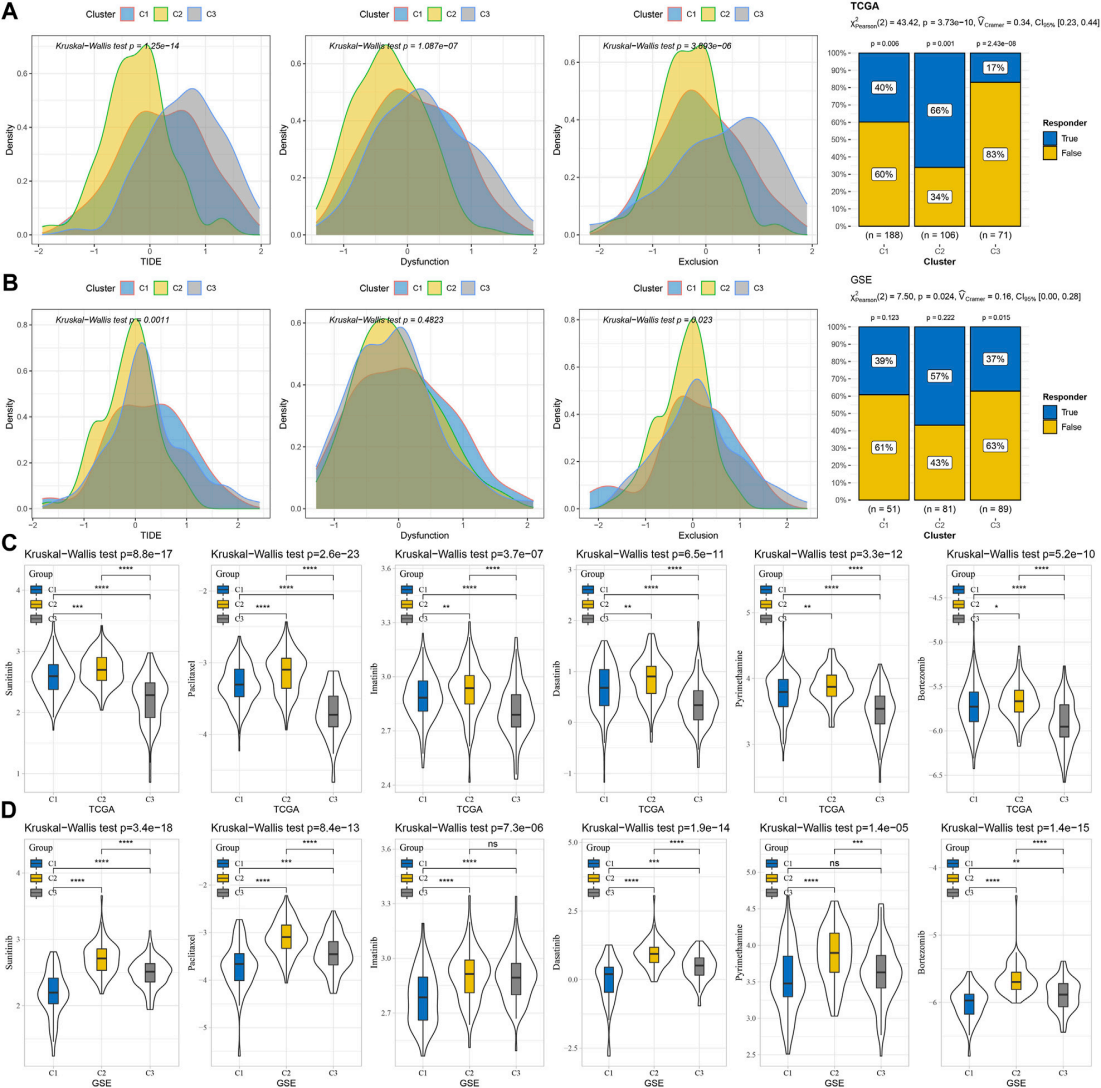
Different responses of three macrophage-related subtypes to immunotherapy and anti-tumor drugs. **(A)** TIDE score, dysfunction score, and exclusion score distribution and response rate to immunotherapy of three macrophage-related subtypes in TCGA-LIHC dataset. **(B)** The distribution of the TIDE score, dysfunction score, and exclusion score and the response rate to immunotherapy of the three macrophage-related subtypes in the GSE14520 cohort. **(C)** IC_50_ values of sunitinib, paclitaxel, imatinib, dasatinib, pyrimethamine, and bortezomib in the three macrophage-related subtypes of the TCGA-LIHC dataset. **(D)** The sensitivity of sunitinib, paclitaxel, imatinib, dasatinib, pyrimethamine, and bortezomib in the three macrophage-related subtypes of the GSE14520 cohort. The significance of the difference was marked with *, *p < 0.05, **p < 0.01, ***p < 0.001, ****p < 0.0001, and ns, no difference.

To screen the subtypes that were expected to be more suitable for immunotherapy from the three macrophage-related subtypes, TIDE was used to calculate the TIDE score and the potential response rate to immunotherapy based on the RNA-seq data of three macrophage-related subtypes in TCGA-LIHC and GSE14520. The three macrophage-related subtypes in TCGA-LIHC showed significant differences in the TIDE score, dysfunction score, and exclusion score distribution and response rate to immunotherapy. Compared to C1 (40%) and C3 (17%), C2 had a much greater response rate to immunotherapy at 66% (Figure 5A). The three macrophage-related subgroups in the GSE14520 cohort showed significant variations in the TIDE score, exclusion score distribution, and immunotherapy response rate. The response rate of C2 to immunotherapy in this cohort was also the highest among the three macrophage-related subtypes and was significantly higher than that of C1 and C3 (Figure 5B). Although C2 was most suitable for immunotherapy, this subtype was the most resistant subtype of the three macrophage-related subtypes to sunitinib, paclitaxel, imatinib, dasatinib, pyrimethamine, and bortezomib (Figures 5C, D).

### Construction and verification of the risk system consisting of important macrophage-specifically highly expressed genes

**FIGURE 6:**
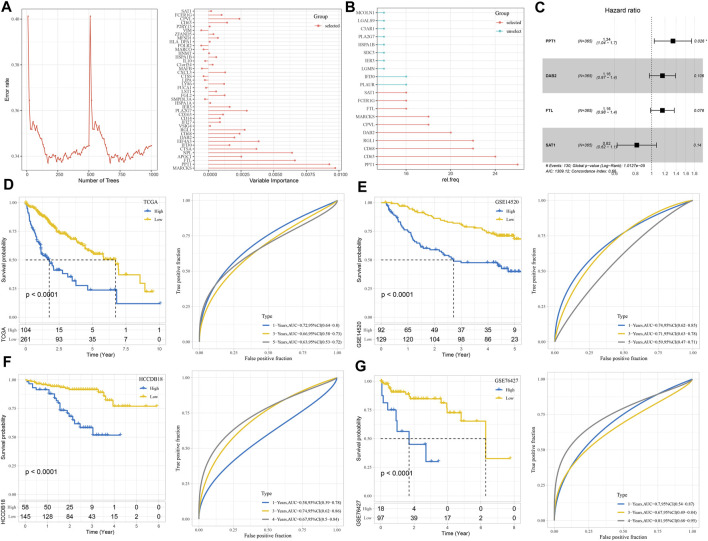
Construction and verification of the risk system consisting of important macrophage marker genes. **(A)** The relationship between the number of classification trees and error rate and the out-of-bag feature importance of macrophage marker genes. **(B)** Random survival forest variable hunting analysis. **(C)** Multivariate Cox regression forest map of four macrophage marker genes in the risk system. **(D–G)** The prognostic predictive performance of the risk system was evaluated in TCGA-LIHC, GSE14520, HCCDB18, and GSE76427 cohorts: Kaplan–Meier survival curve and ROC curve.

The random forest algorithm was utilized to carry out macrophage marker gene selection, and the out-of-bag error was used as an index to quantify the classification error and evaluate performance. A total of 10 important specifically highly expressed genes, namely, STA1, FCER1G, FTL, MARCKS, CPVL, DAB2, RGL1, CD68, CD63, and PPT1, were selected according to the relationship between the error rate and the number of classification trees and the out-of-bag feature importance of macrophage-specifically highly expressed genes (Figures 6A, B). Stepwise multivariate regression analysis realized the development of a risk system according to the following formula: risk score = 0.293× PPT1 + 0.149× DAB2 +0.148× FTL-0.204× SAT1 (Figure 6C). Using this risk system, each sample in the TCGA-LIHC cohort, GSE14520 cohort, HCCDB18 cohort, and GSE76427 cohort was assigned a risk score, and a higher risk score was significantly associated with a poor prognosis in each cohort. However, the time at which the risk system maximizes the accuracy of survival prediction was different in different cohorts. For example, the performance of predicting 1-year overall survival (OS) in the TCGA-LIHC cohort and GSE14520 cohort was the best, and the AUC of ROC was 0.72 and 0.74, respectively. The prediction accuracy of the risk system for 3-year OS of the HCCDB18 cohort was the highest, reaching 0.74, while the prediction accuracy of 5-year OS (AUC = 0.81) of the GSE76427 cohort was much higher than that of 1-year (AUC = 0.7) and 3-year OS (AUC = 0.67) (Figures 6D–G).

### Predictability of the risk model to TME characteristics, immunotherapy response, and drug sensitivity

**FIGURE 7:**
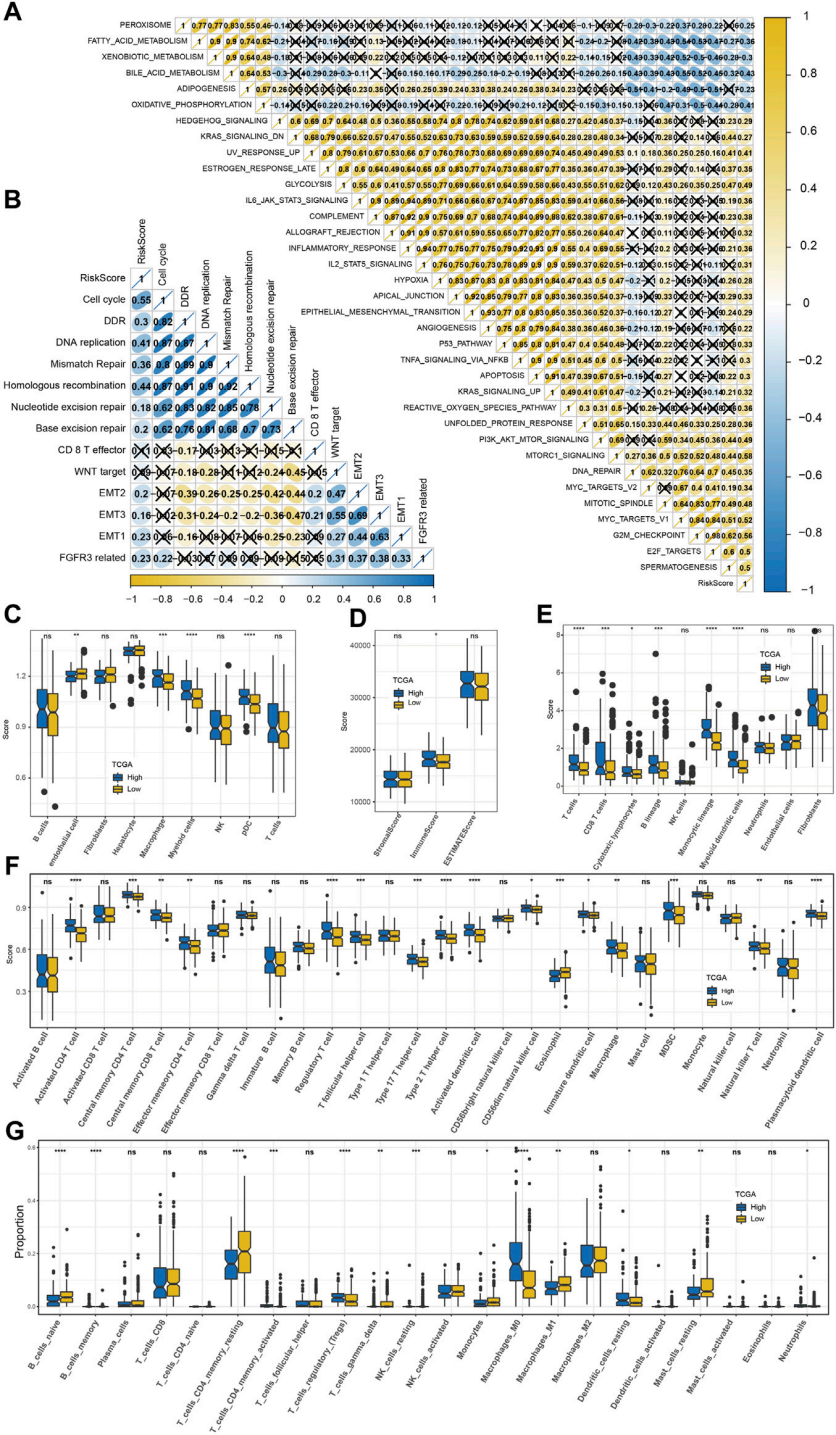
Predictability of the risk model to TME characteristics. **(A)** The correlation between the risk score and 35 pathways that showed significant differences between the high-risk group and low-risk group (X means *p* > 0.05). **(B)** The association between the risk score and 13 core biological pathways (X means *p* > 0.05). **(C)** Differences in the distribution of nine kinds of cells among three macrophage-related subtypes identified by scRNA-seq analysis. **(D)** ESTIMATE analysis. **(E)** MCP-counter analysis. **(F)** ssGSEA analysis. **(G)** CIBERSORT analysis. The significance of the difference was marked with *, *p < 0.05, **p < 0.01, ***p < 0.001, ****p < 0.0001, and ns, no difference.

**FIGURE 8:**
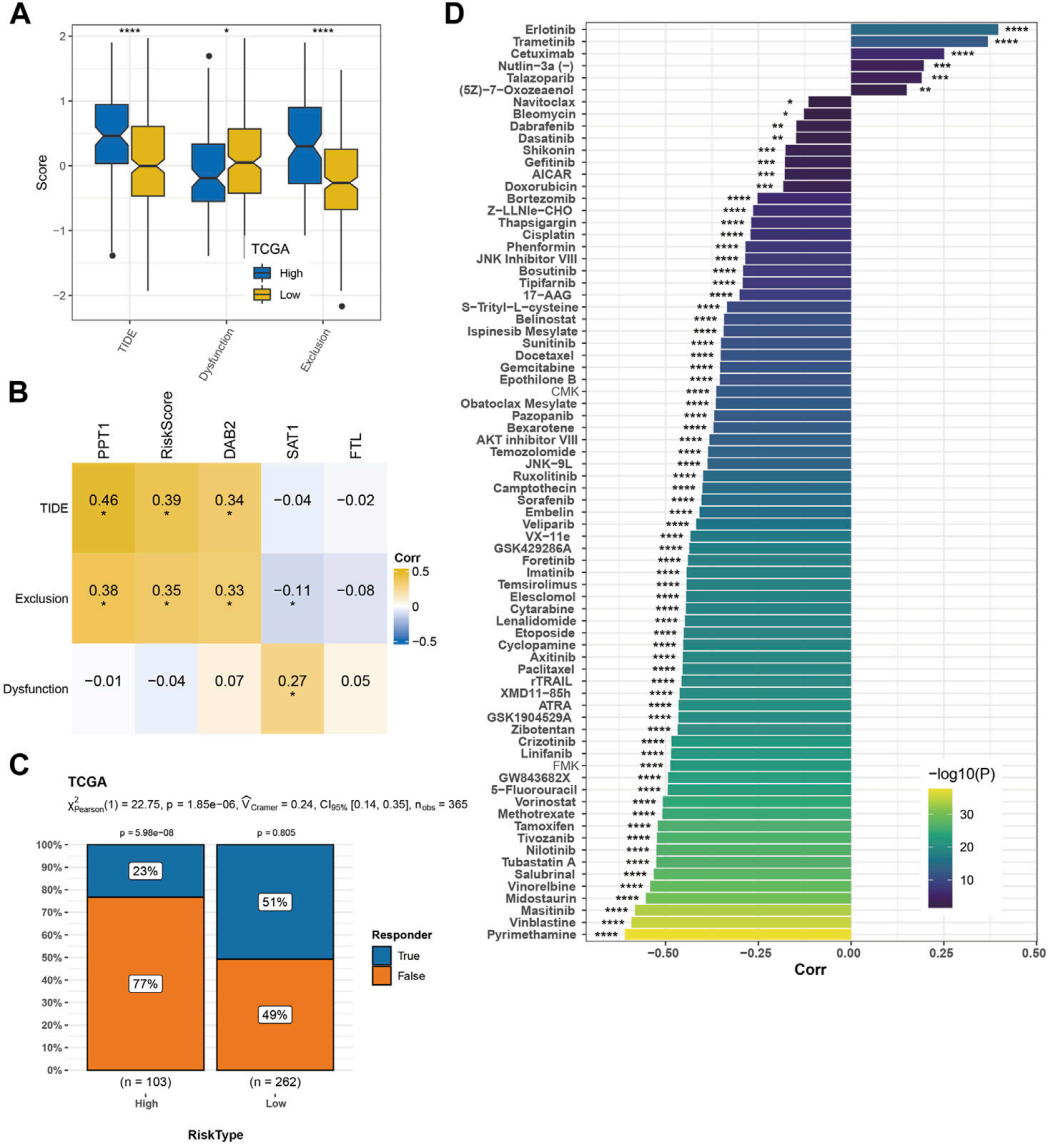
Predictability of the risk model to immunotherapy response and drug sensitivity. **(A)** TIDE score, dysfunction score, and exclusion score of high-risk and low-risk groups. **(B)** Pearson’s correlation analysis of macrophage marker genes and risk score in the risk system with TIDE score, dysfunction score, and exclusion score. **(C)** The response rate of the two risk groups to immunotherapy. **(D)** Drug sensitivity prediction. The significance of the difference was marked with *, *p < 0.05, **p < 0.01, ***p < 0.001, and ****p < 0.0001.

We found 35 pathways showing significant differences between the two risk groups. The risk score was positively correlated with immune and carcinogenic pathways but negatively correlated with metabolic pathways in these 35 pathways, including oxidative phosphorylation, fatty acid metabolism, peroxisome, bile acid metabolism, and xenobiotic metabolism (Figure 7A). The risk score was highly positively correlated with the cell cycle, DNA damage response (DDR), DNA replication, mismatch repair, and homologous recombination (Figure 7B). Among the nine kinds of cells that comprise HCC identified by scRNA-seq analysis, the content of endothelial cells in the low-risk sample was significantly higher than that in the high-risk sample, while the content of macrophages, myeloid cells, and pDCs was significantly increased in the high-risk sample compared with the low-risk sample (Figure 7C). The immune score, immune cells including B lineage, myeloid dendritic cells, activated/central memory CD4 T cells, T cells, monocytic lineage, CD8 T cells, CD4 T cells, macrophage, regulatory T cells, cytotoxic lymphocytes, and activated dendritic cells had significantly higher abundances in high-risk samples than in low-risk samples (Figures 7D–F). CIBERSORT analysis not only validated the B- and T-cell differences but also distinguished the macrophage subtype discrepancies between the two risk groups. Higher proportions of macrophage_M0 and lower proportions of macrophage_M1 were observed in the high-risk group, while the low-risk group displayed the opposite phenomenon (Figure 7G). TME characteristics were executed in two risk groups of the GSE14520 cohort with analogous observations (Supplementary Figure S4). Moreover, the high-risk group was significantly relevant to the high TIDE score, exclusion score, and low dysfunction score (Figure 8A). Macrophage-specifically highly expressed genes PPT1 and DAB2 and risk score in the risk system showed a significant positive correlation with the TIDE score and exclusion score, while SAT1 showed significant negative correlation with the dysfunction score (Figure 8B). There was a very significant difference in the response rate to immunotherapy between the two risk groups, with a potential response rate of 51% in the low-risk group and 23% in the low-risk group (Figure 8C). The correlation analysis between the risk score and the sensitivity of anticancer drugs showed that the risk score was significantly linked with the sensitivity of most drugs such as pyrimethamine, vinblastine, and masitinib (Figure 8D).

### Predictability of the risk model for prognosis and immunotherapy responses in different immunotherapy cohorts

**FIGURE 9:**
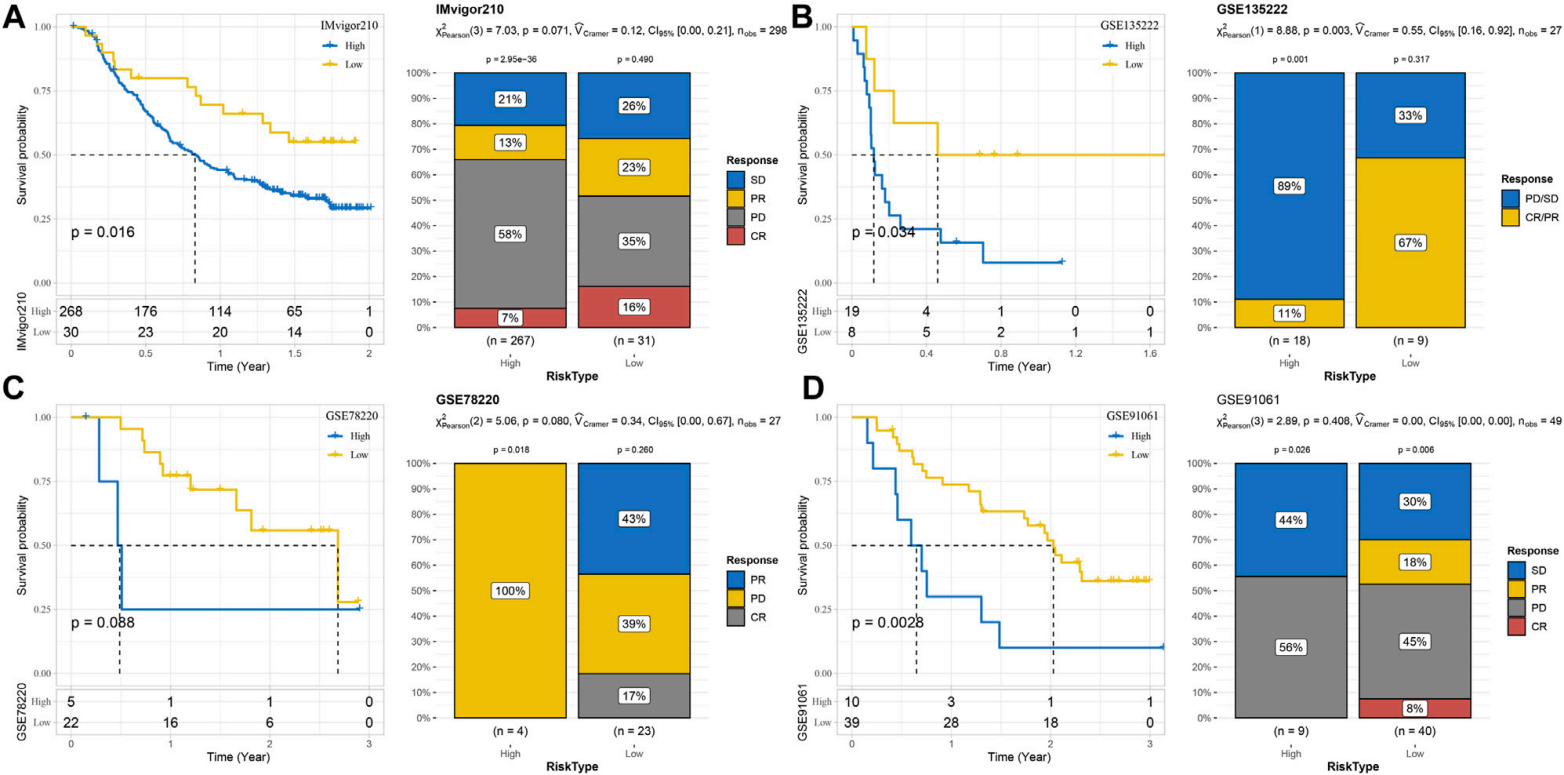
Predictability of the risk model for prognosis and immunotherapy responses in different immunotherapy cohorts. **(A)** Risk model assesses prognosis and immunotherapy response in the IMvigor210 cohort. **(B)** The prognosis and immunotherapy response rate of samples in the GSE135222 cohort were predicted according to the risk model. **(C)** The risk model predicted the outcome of prognosis and immunotherapy response in the GSE78220 cohort. **(D)** Discrimination of patient prognosis and immunotherapy response by the risk model in the GSE91061 dataset.

Survival analysis and immunotherapy response assessment were performed in four immunotherapy cohorts according to the risk system. The risk system significantly distinguished the 2-year survival rate between high-risk and low-risk samples in the IMvigor210 cohort. Responses to immunotherapy comprised four conditions: complete response (Becht et al., 2016), partial response (Becht et al., 2016), stable disease (Liberzon et al., 2015), and progressive disease (PD). Here, 39% of the low-risk group responded to immunotherapy, while 20% of the high-risk group responded to immunotherapy (Figure 9A). The OS of the GSE135222 cohort was also differentiated under the calculation of the risk model, and the difference in response rates to immunotherapy was significant between the two risk groups (low vs. high = 67% vs. 11%) (Figure 9B). The risk model failed to significantly predict survival and immunotherapy differences in the GSE78220 cohort, with all samples in the high-risk group progressing to PD and 17% of those in the low-risk group achieving CR to immunotherapy (Figure 9C). The risk model could significantly identify the difference in the 3-year prognosis of patients in the GSE91061 dataset but could not significantly distinguish the difference in immunotherapy response between the two risk groups (Figure 9D).

### Validation of the expression of four model genes in HCC cells

**FIGURE 10:**
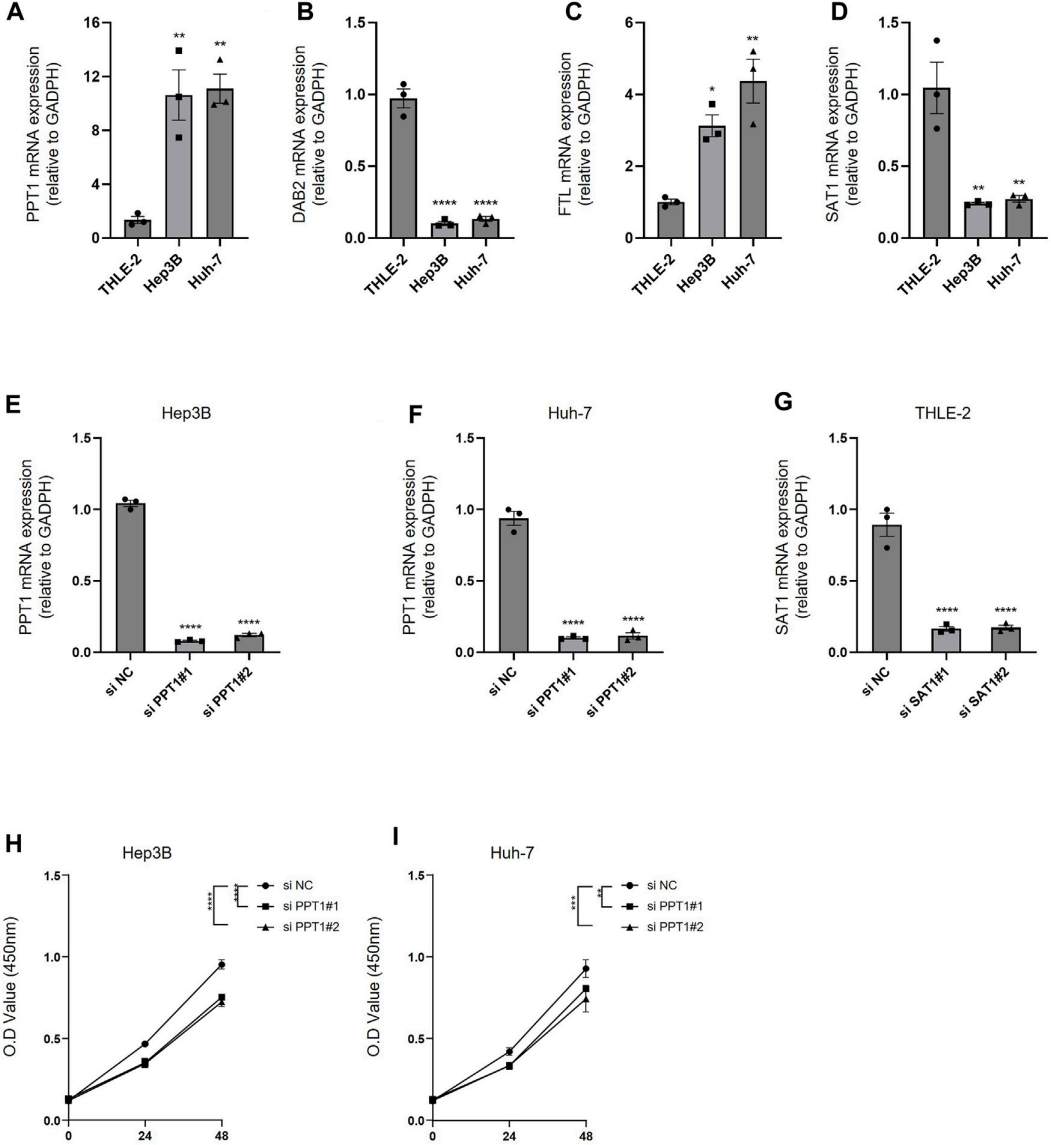
Experimental validation of predictive models. **(A–D)** PCR was performed to detect the expression of PPT1, DAB2, FTL, and SAT1 in THLE-2, Hep3B, and Huh-7 cells. **(E–F)** The inhibitory efficiency of si PPT1 was verified in Hep3B and Huh-7 cell lines. **(G)** The inhibitory efficiency of si SAT1 was verified in THEL-2 cells. **(H)** Alterations in cell viability following inhibition of PPT1 expression in Hep3B cells. **(I)** Alterations in cell viability following inhibition of PPT1 expression in Huh-7 cells. N = 3. The significance of the difference was marked with *, *p < 0.05, **p < 0.01, ***p < 0.001, and ****p < 0.0001. The results are presented as the mean ± SEM.

To verify the efficacy of our predictive model, we validated the expression of PPT1, DAB2, FTL, and SAT1 using PCR in HCC cells (Hep3B and Huh7), as well as human normal hepatocytes (THLE-2). We found that PPT1 and FTL were highly expressed in hepatocellular carcinoma cell lines, while DAB2 and SAT1 were highly expressed in human hepatocytes (Figures 10A–D). We then used siRNA to inhibit the expression of PPT1 in HCC cell lines and SAT1 in human hepatocytes THLE-2. The results of PCR assay showed that the siRNA possessed good transfection efficiency (Figures 10E–G). We then verified the cell viability of HCC cell lines after inhibition of PPT1 in Hep3B and Huh7 cell lines using CCK8 experiments. The results displayed that cell viability decreased after inhibition of PPT1 (Figures 10H, I). All the aforementioned results validate the reliability of our prediction model.

## Discussion

HCC is a common liver disease, and its progression is regulated by the immune system (Donne and Lujambio, 2023). The main cellular components of HCC include cancer cells, immune cells, and stromal cells, and the relationship between different cell types and clinical relevance of HCC is not clear (Arvanitakis et al., 2023). Immune cells are considered to be the main contributors to tumor immunosuppression, anti-tumor drug resistance, and tumor clearance. T cells (36%) accounted for the highest proportion of all immune cell types in HCC, followed by NK cells (29%) and macrophages (25%) (Zhang et al., 2022). In this study, we found that HCC was composed of nine types of cells through scRNA-seq analysis, with hepatocytes, T cells, and macrophages accounting for the highest proportion. The sequencing results of a previous study showed that specific TAMs are a hub node that connects different cell groups in the cell–cell interaction network and can regulate tumorigenesis and anti-tumor immunity (Zhou et al., 2021). In this study, through the analysis of the communication between nine kinds of cells, we found that the cell with the highest intensity of communication with B cells, endothelial cells, myeloid cells, NK cells, and pDCs was macrophages. At the same time, macrophage was also one of the cells with the strongest communication with fibroblasts, hepatocytes, and T cells, which also reflected the hub role of macrophage in HCC.

There is important evidence that TAM-based immune classification may provide tools for customized chemotherapy and immunotherapy (Sun et al., 2022). In this study, the subtype of HCC was defined according to the marker genes of macrophage. We identified 56 prognosis-related genes in the 179 specifically highly expressed genes of macrophage and classified HCC into three subtypes according to their level in the transcriptome. The subtypes with the best and worst prognoses were C2 and C3, respectively. Indicators related to immunotherapy response, including pTMB and TIDE score, showed significant differences between C2 with the best prognosis and C3 with the worst prognosis. C2 showed the highest pTMB and response rate of immunotherapy and the lowest TIDE score, which indicated that C2 was the most suitable subtype of immunotherapy among the three subtypes. For C3 with the worst prognosis, we found that this subtype was more suitable for targeted therapy and chemotherapy than C2 and was sensitive to sunitinib, paclitaxel, dasatinib, pyrimethamine, and bortezomib.

Identifying specific macrophage markers to design targeted and personalized drugs is essential for the prevention and treatment of malignant liver tumors (Cheng et al., 2022). The effectiveness of macrophage markers as a strategy for designing patient prognostic gene classifiers has been applied in a variety of cancers, such as glioma (Sun et al., 2019), prostate cancer (Siefert et al., 2021), bladder cancer (Jiang et al., 2022), and triple-negative breast cancer (Bao et al., 2021). In this study, Cox regression analysis and random survival forest analysis were applied to select four genes from 179 specifically highly expressed genes of macrophage to achieve the development of the risk system. Among them, PPT1 (*Palmitoyl protein thioesterase 1*) is highly expressed in HCC tissues, especially in macrophages. The research further revealed that HCC patients with low intra-tumoral PPT1^+^ macrophage infiltration tend to have a survival advantage, indicating that targeting PPT1 may serve as an immunotherapeutic biomarker in HCC (Weng et al., 2023). DAB2 (*disabled-2*) is highly expressed in tumor-infiltrating TAM, and its genetic ablation can significantly damage the formation of lung metastasis. DAB2 is associated with poor prognosis of human lobular breast and gastric carcinomas (Marigo et al., 2020). Furthermore, the overexpression of DAB2 eliminated the effectiveness of dendritic cell vaccines in the context of dendritic cell-relevant tumor immunotherapy (Ahmed et al., 2015). Hypoxia-inducible FTL (*ferritin light chain*), one of the hub ferroptosis regulators (Yan et al., 2023), functions as a new biomarker for the responsiveness to temozolomide in glioblastoma, as well as a prognostic marker (Liu et al., 2020a). SAT1 (*spermidine/spermine-N1-acetyltransferase 1*) was chosen as a protective factor to construct a ferroptosis-relevant prediction model in HCC patients (Wang et al., 2021). Further research demonstrated that the expression of SAT1 was repressed in HCC tumor tissues compared with normal liver tissues (Long et al., 2023), which was also demonstrated in our validation test. The tumor-suppressor protein, p53, was discovered to have the ability to induce ferroptosis and inhibit tumor growth through facilitating SAT1 expression (Liu et al., 2020b). In this research, these four genes may be treated as novel marker genes of macrophage in HCC. In addition, PPT1 and DAB2 may also serve as new markers for immunotherapy.

In this study, our analysis showed that the risk system integrated with these four genes showed accuracy and reliability in predicting OS in all four HCC cohorts, and it also helped screen patients suitable for immunotherapy and predict the sensitivity of some targeted drugs and chemical drugs to patients, which may be beneficial to the choice of personalized treatment options for patients.

To sum up, the current study applied scRNA-seq analysis to determine nine types of cells in HCC and to identify the core role of macrophage in HCC. Moreover, three HCC models with different prognoses, TME, and immunotherapy response levels were defined according to specifically highly expressed genes in macrophages, and a risk system based on the aforementioned macrophage genes was constructed, which provided a new insight into the prognosis target and preclinical personalized treatment choice of HCC.

## Data Availability

The original contributions presented in the study are included in the article/Supplementary Material; further inquiries can be directed to the corresponding author.

## Abbreviations

HCC, hepatocellular carcinoma; TME, tumor microenvironment; TAM, tumor-associated macrophage; scRNA-seq, single-cell RNA sequencing; GEO, Gene Expression Omnibus; TCGA, The Cancer Genome Atlas; UMI, unique molecular identifier; MT, mitochondrial; HVG, highly variable gene; PCA, principal component analysis; CDF, cumulative distribution function; ssGSEA, single-sample gene set enrichment analysis; MSigDB, Molecular Signatures Database; TIDE, Tumor Immune Dysfunction and Exclusion; pDC, plasmacytoid dendritic cell; NK cell, natural killer cell; DDR, DNA damage response; CR, complete response; PR, partial response; SD, stable disease; PD, progressive disease; PPT1, palmitoyl protein thioesterase 1; DAB2, disabled-2; FTL, ferritin light chain; SAT1, spermidine/spermine-N1-acetyltransferase 1; and qRT-PCR, quantitative real-time polymerase chain reaction.
