# Supplementary figures and images for: The core role of macrophages in hepatocellular carcinoma: the definition of molecular subtypes and the prognostic risk system
Source: Front Pharmacol. 2023 Aug 24;14:1228052. doi: 10.3389/fphar.2023.1228052 (PMC10491020; doi:10.3389/fphar.2023.1228052)

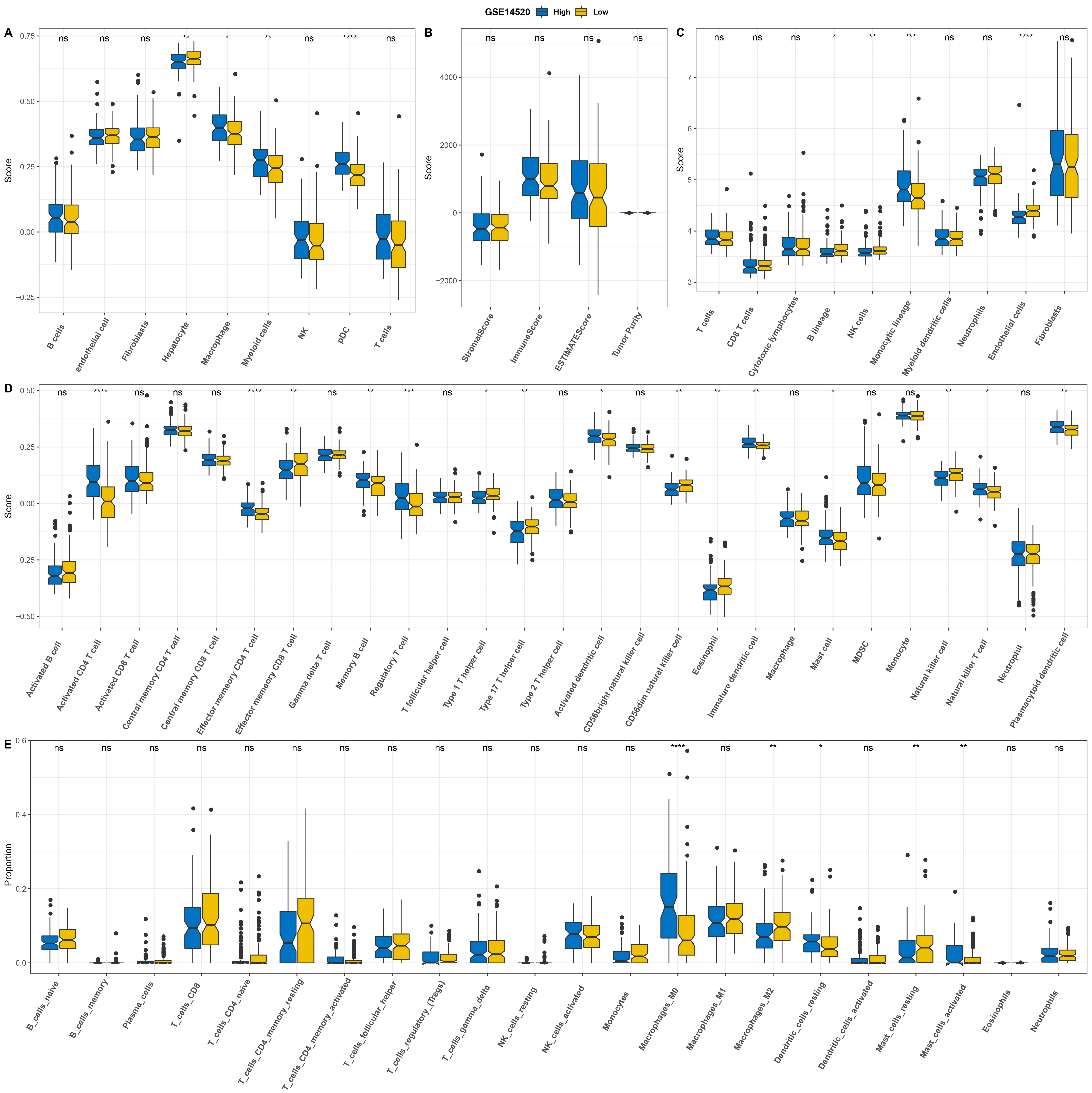

Supplement: Supplementary file 1 [file Image4.JPEG]

# TCGA

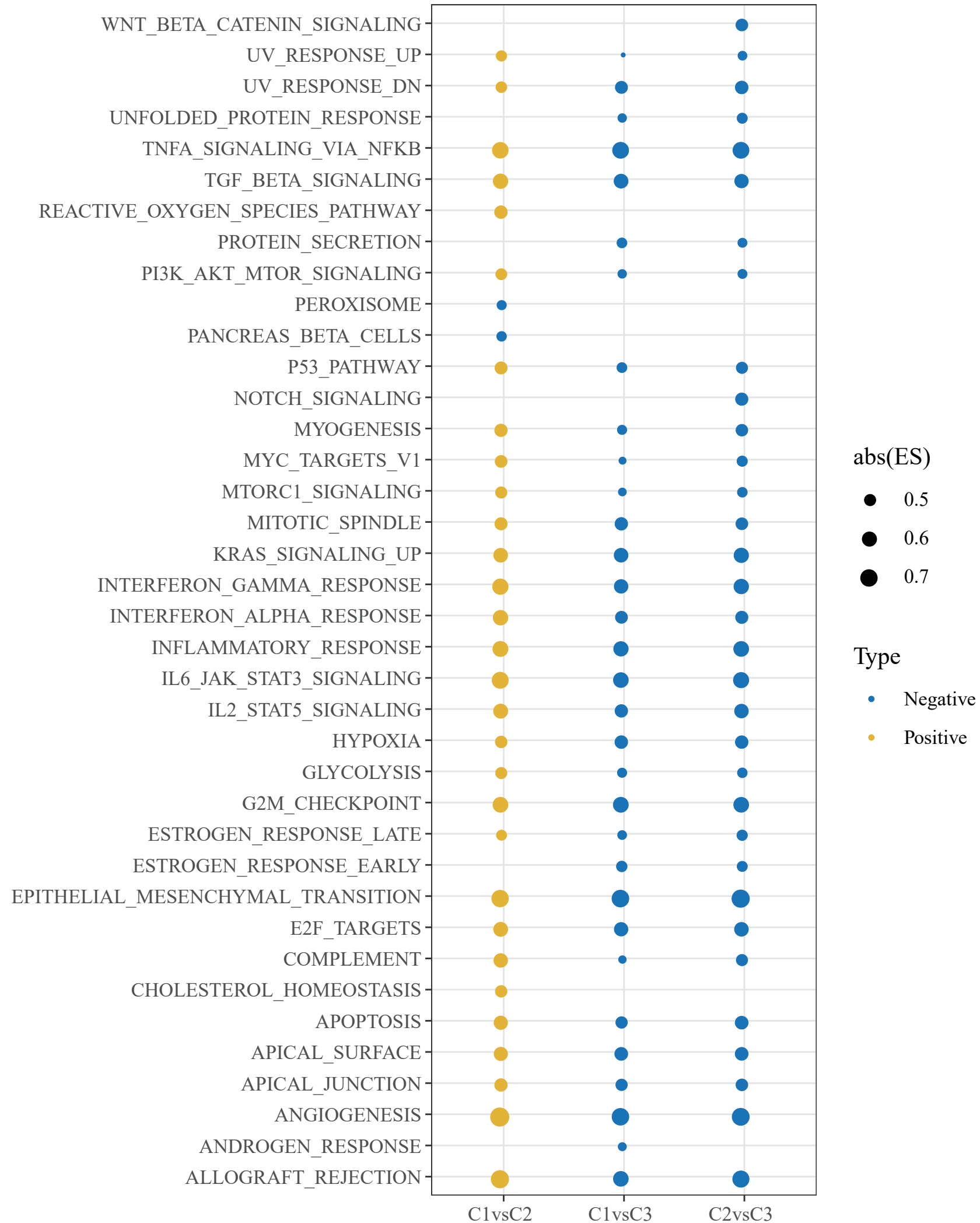

Supplement: Supplementary file 2 [file Image2.PDF]

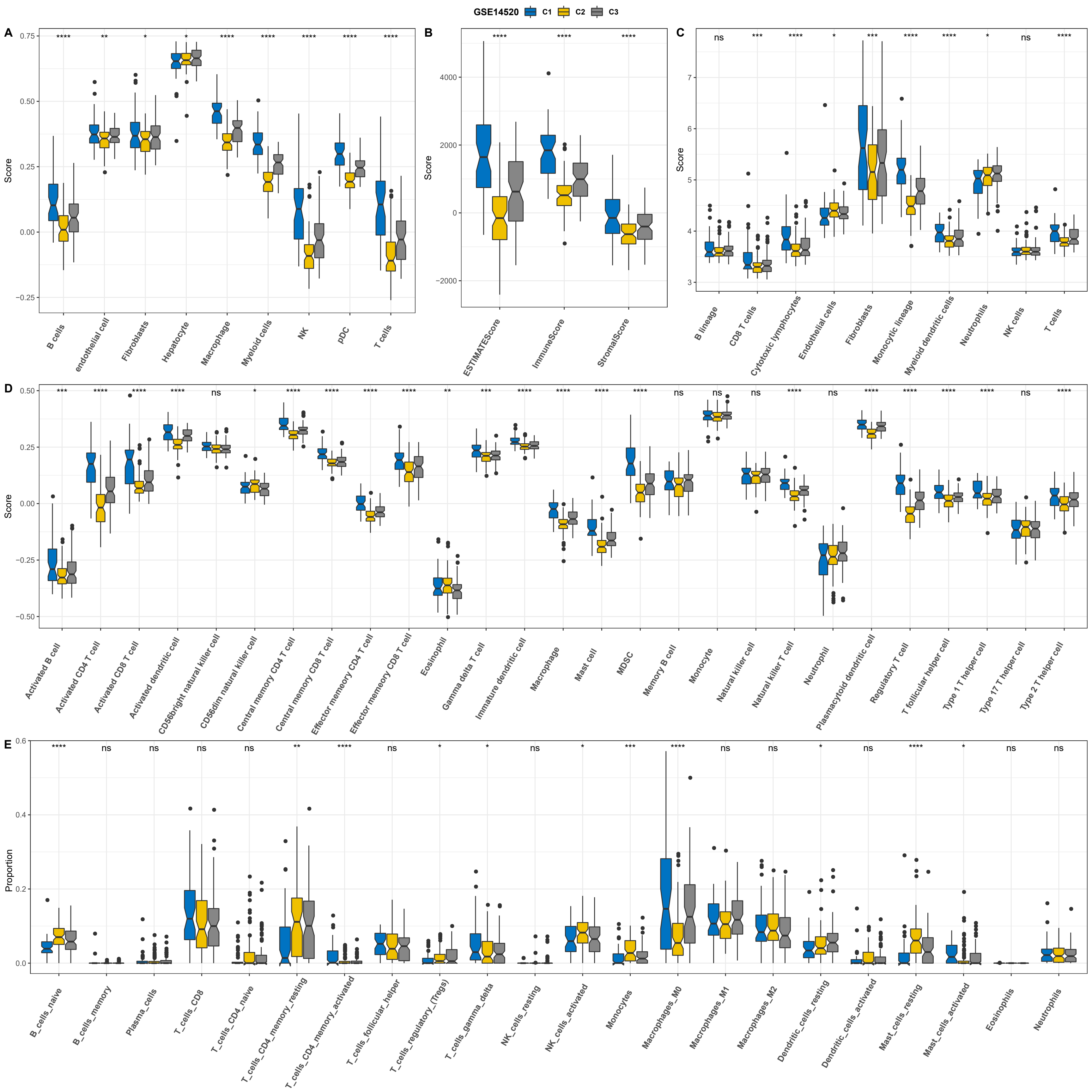

Supplement: Supplementary file 3 [file Image3.PDF]

A

Number of interactions

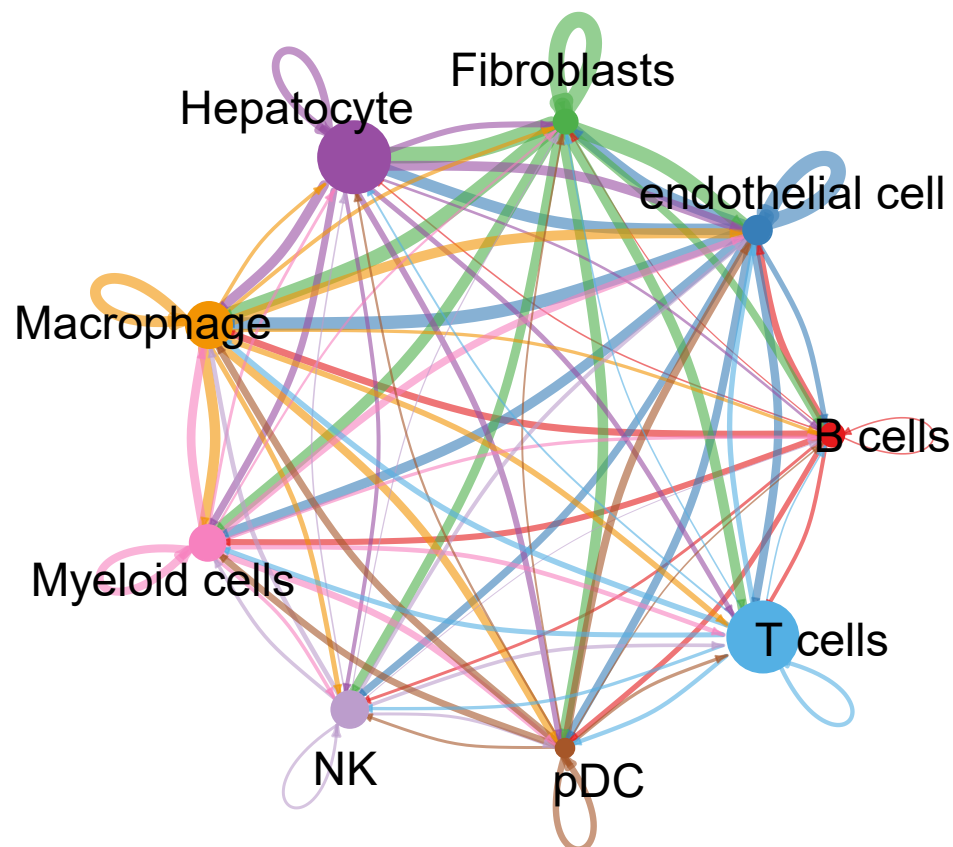

B

Interaction weights/strength

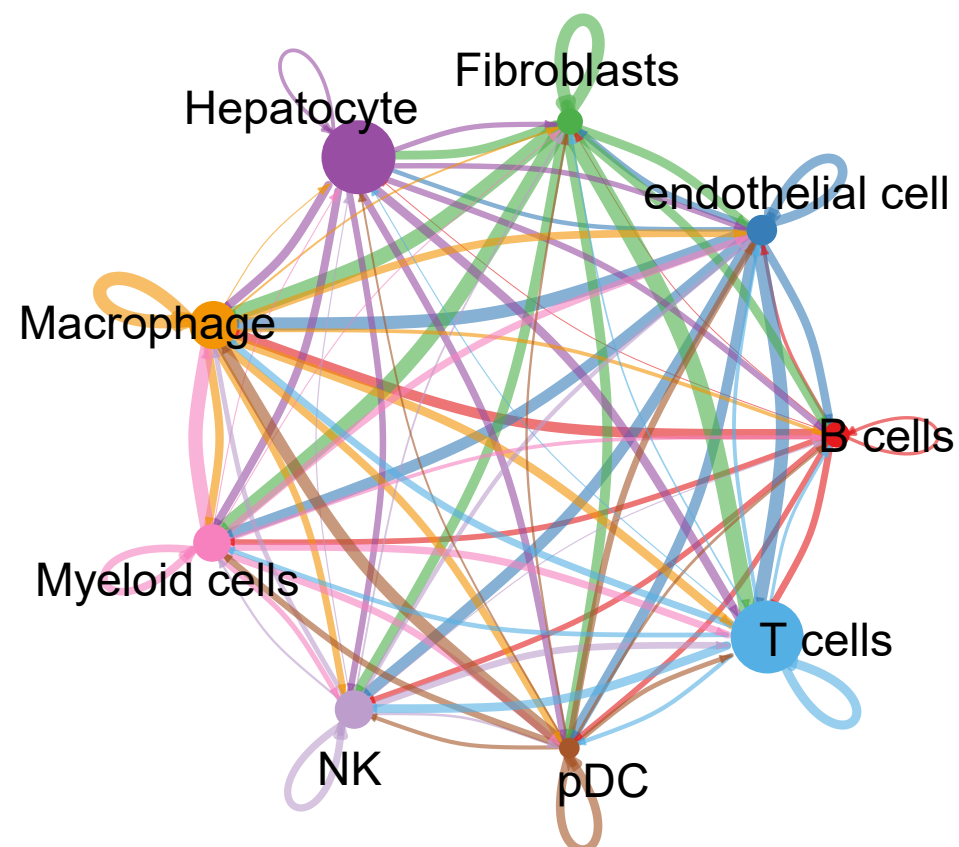

C

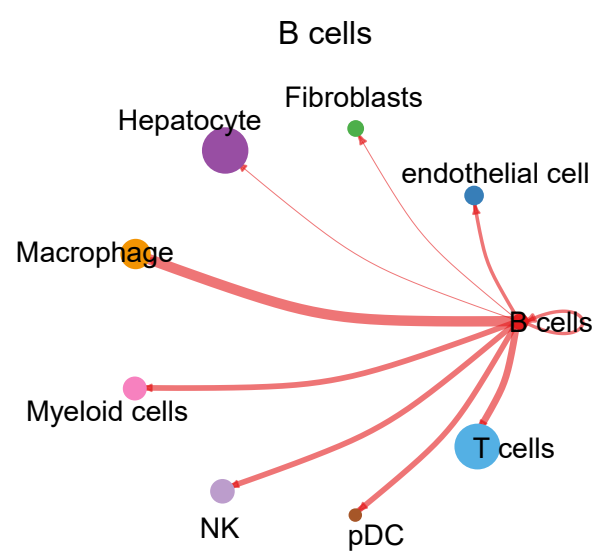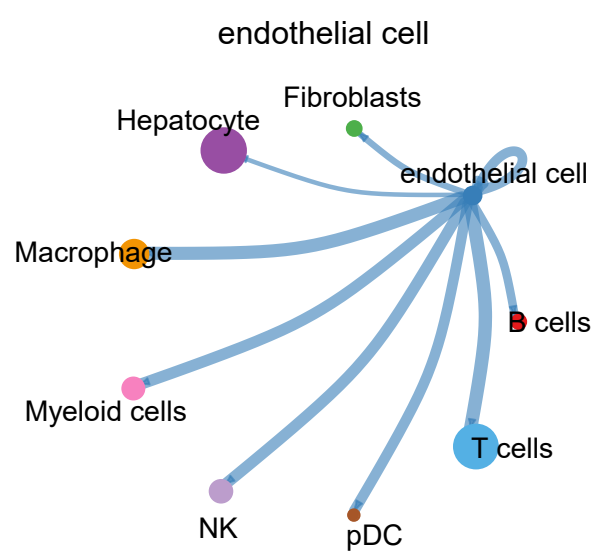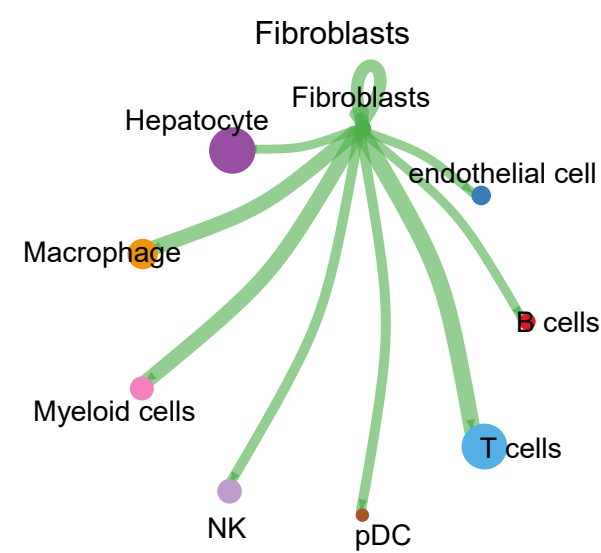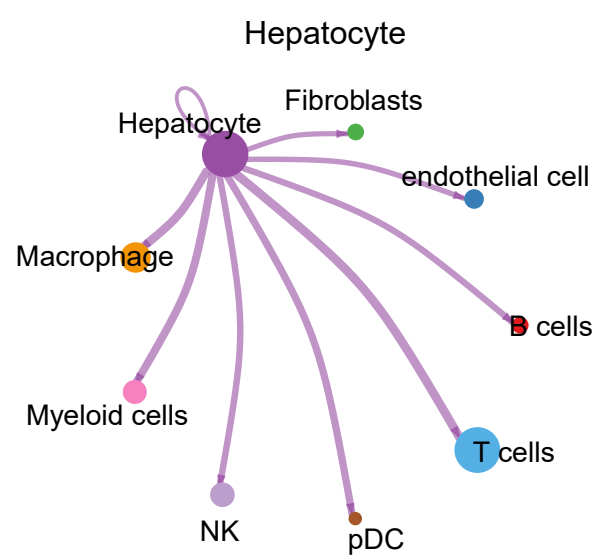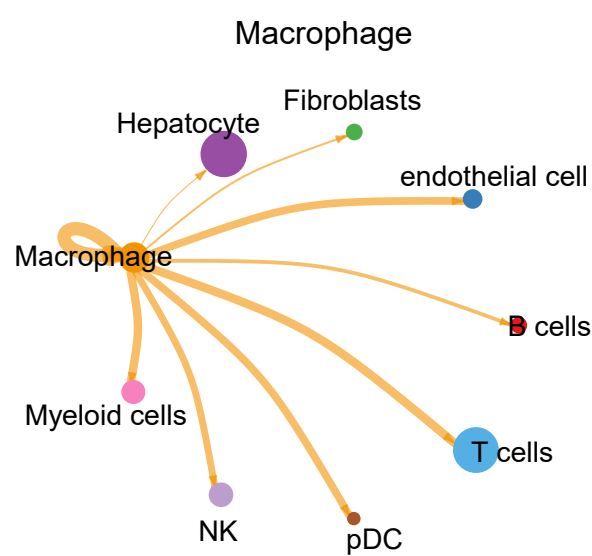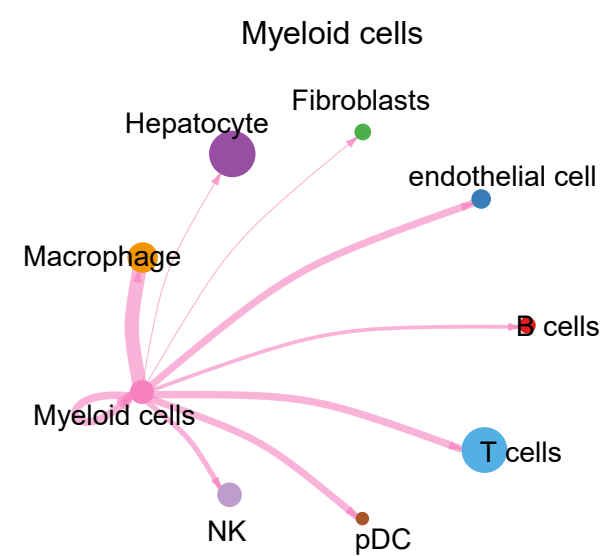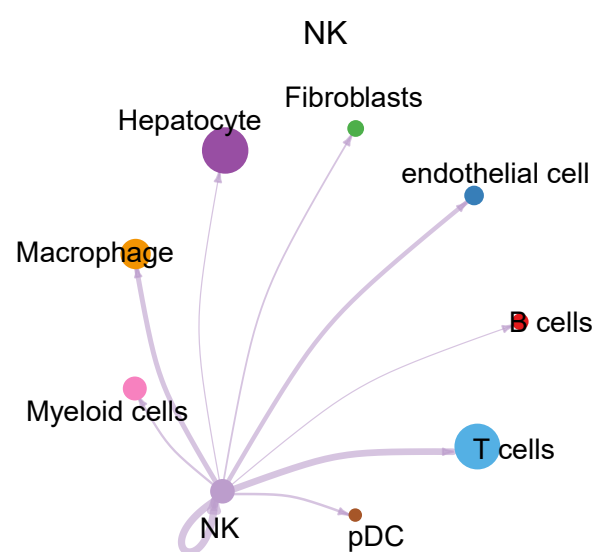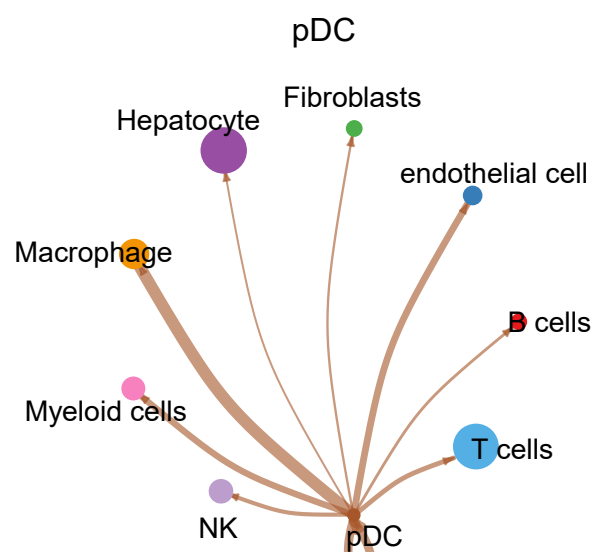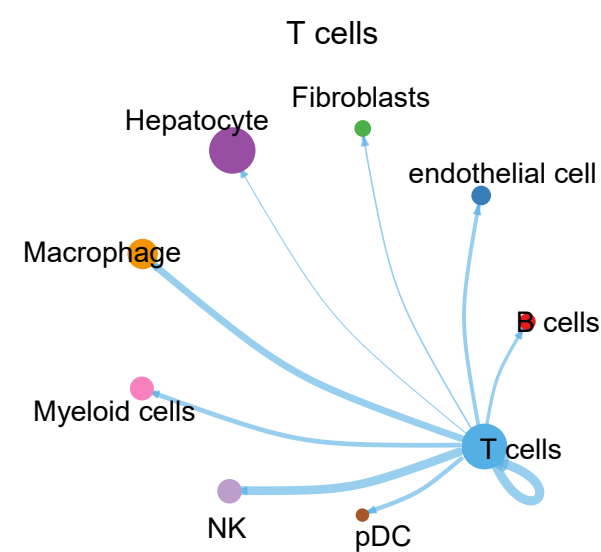

Supplement: Supplementary file 5 [file Image1.PDF]
