# Supplementary material for: The core role of macrophages in hepatocellular carcinoma: the definition of molecular subtypes and the prognostic risk system
Source: Front Pharmacol. 2023 Aug 24;14:1228052. doi: 10.3389/fphar.2023.1228052 (PMC10491020; doi:10.3389/fphar.2023.1228052)
Supplement: Supplementary file 4 [file DataSheet1.docx]

Legends of Supplementary Figures

**Supplementary Figure 2 Enrichment differences of different biological pathways among subtypes**

**Supplementary Figure 3 Immunological features of three macrophage related subtypes in GSE14520 database**

(A) The differences in the distribution of 9 kinds of cells among three macrophage related subtypes identified by scRNA-seq analysis. (B) ESTIMATE analysis. (C) MCP-counter analysis. (D) ssGSEA analysis. (E) CIBERSORT analysis.

The significance of the difference was marked with *, *p<0.05, **p<0.01, ***p<0.001, ****p<0.0001, ns, no difference.

**Supplementary Figure 4 Immunological features of the riskscore signature in GSE14520 database**

(A) The differences in the distribution of 9 kinds of cells among three macrophage related subtypes identified by scRNA-seq analysis. (B) ESTIMATE analysis. (C) MCP-counter analysis. (D) ssGSEA analysis. (E) CIBERSORT analysis.

The significance of the difference was marked with *, *p<0.05, **p<0.01, ***p<0.001, ****p<0.0001, ns, no difference.
